# Dentists in the Army

**Published:** 1899-05

**Authors:** Geo. W. Griswold


ARTICLE VIII.

### DENTISTS IN THE ARMY.

GEO. W. GRISWOLD, D. D. S.

The subject is one that has been discussed at some length by the dental brethren, and I will not attempt any long-winded argument in regard to it; but from my recent experience in the volunteer forces of the United States I might possibly be able to give some information on the subject.

The soldier's life is one that requires a sound body, a sound mind, and a sound set of teeth, and the average soldier is, I believe, desirous of taking good care of his teeth, for he realizes their importance, when it comes to masticating Uncle Sam's hard-tack, salt pork, bacon, etc. But how is he going to take good care of them? On the salary he is getting he cannot afford "dentists' bills," and if he could, he is only allowed to leave camp or garrison at long intervals, and feels that his leave is too previous to be spent in a dentist's chair.

The result is that his teeth are neglected and decay progresses until some fine day when blissfully eating his rations a piece of hard-tack gets into a cavity, crushes through to the nerve, causing him to jump four feet in the air, and yelling like a Comanche Indian, rushes off to the regimental surgeon, who seats him on a camp stool, calmly surveys the offending member, get two or three grinning hospital stewards to hold the victim, while he produces a pair of antiquated "archaic" weapons that some dealer in surgical instruments has palmed off on him as a universal forcep, grabs the tooth, and, placing his knee on the patient's chest to give him a purchase, gives a long, steady, straight pull that would not bring the tooth in a hundred years, and smash she goes. The result is that the next time that particular man or his tentmates have toothache they prefer to stick it out rather than be butchered, and consequently are unfit for duty.

How much better it would be if every regiment had its dentist, a man who has been in continuous practice long enough to have passed the "smart aleck" stage. Such a man, with a capable assistant, could by frequent inspections and a strict attention to business help wonderfully from a physical standpoint to improve the army and naval service of the United States.—*Dental Review.*

